# Suppression of beta oscillations in the subthalamic nucleus following cortical stimulation in humans

**DOI:** 10.1111/j.1460-9568.2008.06363.x

**Published:** 2008-10

**Authors:** L M F Doyle Gaynor, A A Kühn, M Dileone, V Litvak, A Eusebio, A Pogosyan, A G Androulidakis, S Tisch, P Limousin, A Insola, P Mazzone, V Di Lazzaro, P Brown

**Affiliations:** 1 Sobell Department of Motor Neuroscience and Movement Disorders, Institute of Neurology8–11, Queen Square, London WC1N 3BG, UK; 2 Department of Neurology, Charité Campus Virchow, Humboldt UniversityBerlin, Germany; 3 Functional and Stereotactic Neurosurgery, CTO, ASL RMCRome, Italy; 4 Unit of Functional Neurosurgery, Institute of NeurologyLondon, UK; 5 Neurology Department, Catholic UniversityRome, Italy

**Keywords:** basal ganglia, event-related desynchronization, motor cortex, Parkinson’s disease, transcranial magnetic stimulation

## Abstract

It is unclear how subthalamic nucleus activity is modulated by the cerebral cortex. Here we investigate the effect of transcranial magnetic stimulation (TMS) of the cortex on oscillatory subthalamic local field potential activity in the 8–35 Hz (alpha/beta) band, as exaggerated synchronization in this band is implicated in the pathophysiology of parkinsonism. We studied nine patients with Parkinson’s disease (PD) to test whether cortical stimulation can modulate synchronized oscillations in the human subthalamic nucleus. With patients at rest, single-pulse TMS was delivered every 5 s over each primary motor area and supplementary motor area at intensities of 85–115% resting motor threshold. Subthalamic local field potentials were recorded from deep brain stimulation electrodes implanted into this nucleus for the treatment of PD. Motor cortical stimulation suppressed beta activity in the subthalamic nucleus from ∼0.2 to 0.6 s after TMS (repeated measures anova; main effect of time, *P*<0.01; main effect of side, *P*=0.03), regardless of intensity. TMS over the supplementary motor area also reduced subthalamic beta activity at 95% (*P*=0.05) and 115% resting motor threshold (*P*=0.01). The oscillatory activity decreased to 80 ± 26% of baseline (averaged across sites and stimulation intensities). Suppression with subthreshold stimuli confirmed that these changes were centrally driven and not due to peripheral afference. The results may have implications for mechanisms underlying the reported therapeutic benefits of cortical stimulation.

## Introduction

Animal studies show that the cortex modulates subthalamic nucleus (STN) activity (Parent & Hazrati, 1995; Nambu *et al.*, 2000; Magill *et al.*, 2001), but little is known about functional interactions between these structures in humans. In rats and monkeys, motor cortical stimulation evokes early (< 6 ms) and late (10–30 ms) periods of excitation in the STN, attributed to activation of the hyperdirect cortico-subthalamic pathway and disinhibition through the indirect pathway, respectively (Fujimoto & Kita, 1993; Nambu *et al.*, 2000; Magill *et al.*, 2004). Periods of inhibition are interposed between these excitations (Fujimoto & Kita, 1993; Nambu *et al.*, 2000; Magill *et al.*, 2004), possibly due to initial feedback from the globus pallidus, followed by late ‘cortical disfacilitation’ (Fujimoto & Kita, 1993; Magill *et al.*, 2004). Similarly, Strafella *et al.* (2004) reported that transcranial magnetic stimulation (TMS) of the motor cortex could elicit excitation and long inhibition in STN neurons in patients with Parkinson’s disease (PD).

The cortex can therefore exert a synchronizing influence on STN neurons, but it remains uncertain whether it can impact on physiological and pathological oscillatory synchronization within the basal ganglia. The most striking synchronization occurs over the 8–35 Hz (8–12 Hz alpha; 13–35 Hz beta) band, evident in the coupling of activity of neuronal pairs (Levy *et al.*, 2000; Weinberger *et al.*, 2006), the coupling of neuronal activity to simultaneously recorded local field potentials (Kühn *et al.*, 2005; Weinberger *et al.*, 2006), and oscillations in the local field potential itself (Kühn *et al.*, 2005; Weinberger *et al.*, 2006). Although 8–35 Hz synchrony is suppressed in the basal ganglia before and during motor tasks (Courtemanche *et al.*, 2003; Amirnovin *et al.*, 2004; Kühn *et al.*, 2004; Doyle *et al.*, 2005; Androulidakis *et al.*, 2006, 2007), exaggerated activity within this range is implicated in the bradykinesia and rigidity of PD (Gatev *et al.*, 2006; Uhlhaas & Singer, 2006; Hammond *et al.*, 2007).

Oscillatory activity at both cortical and basal ganglia levels may be coherent at rest (Marsden *et al.*, 2001; Williams *et al.*, 2002; Fogelson *et al.*, 2006), but whether phasic cortical input, such as might accompany voluntary movement, modulates 8–35 Hz activity in the basal ganglia is unknown. This issue is not only of physiological interest but is also of therapeutic relevance for PD, as research has shown that repeated cortical stimulation may partially improve parkinsonism (Canavero *et al.*, 2003; Canavero & Bonicalzi, 2004; Fregni *et al.*, 2005; Pagni *et al.*, 2005; Khedr *et al.*, 2006, 2007; Lefaucheur, 2006; Lomarev *et al.*, 2006). The underlying mechanisms are unclear, but one possibility is that cortical stimulation disrupts pathological oscillatory activity in the basal ganglia (Drouot *et al.*, 2004; Pagni *et al.*, 2005; Wu *et al.*, 2007).

We explored how cortical stimulation might modulate subcortical activity in alert subjects, hypothesizing that such stimulation would also suppress basal ganglia activity in the 8–35 Hz band. To this end, we recorded STN oscillatory activity following single-pulse TMS in patients undergoing implantation of this nucleus for deep brain stimulation (DBS).

## Materials and methods

### Patients and surgery

Nine patients (four females) with PD were studied in the interval between electrode implantation and connection of the DBS leads to the subcutaneous battery. Table 1 gives a summary of the clinical details. All patients gave written consent to take part in this study, which was approved by the joint ethics committee of the National Hospital for Neurology and Neurosurgery and the Institute of Neurology, London and the ethics committees of the Catholic University, Rome, and the Charité, Berlin, in accordance with The Code of Ethics of the World Medical Association (Declaration of Helsinki, 1967).

**Table 1:** Summary of patient details

Case	Age (years)	Sex	Disease duration (years)	Predominant symptoms of PD	Surgical centre	Pre-operative motor UPDRS (off/on levodopa)	Post-operative motor UPDRS (off/on DBS), off levodopa	Medications (daily dose at time of operation)
1	50	F	6	Prolonged off periods, fluctuation	Berlin	57/29	NA	Levodopa 800 mg Pramipexol 0.7 mg Tolcapone 300 mg Amantadine 300 mg Apomorphine 16 mg Domperidone 30 mg
2	66	M	26	Freezing and stiffness	London	39/12	24/11	Levodopa 300 mg Ropinirole 9 mg
3	52	F	17	Freezing and dyskinesias	Rome	52/46	NA	Levodopa 1000 mg Entacapone 800 mg
4	47	M	7	Dyskinesias, tremor, freezing	Rome	32/11	30/13	Levodopa 1100 mg Entacapone 400 mg Cabergoline 2 mg Quetiapine 150 mg
5	69	F	14	Rigidity, gait disturbance, freezing	Rome	29/9	27/11	Levodopa 1250 mg
6	53	M	8	Tremor, gait difficulties	London	26/3	26/13	Ropinirole 12 mg
7	55	M	8	Freezing, rigidity, dyskinesia	Rome	45/35	30/15	Levodopa 825 mg Entacapone 125 mg
8	53	M	10	Dyskinesias	London	23/6	43/10	Levodopa 1000 mg Cabergoline 4 mg
9	58	F	12	Freezing, rigidity, dyskinesia	London	36/10	53/17	Cabergoline 2 mg

NA, 6–12 month post-operative scores not available yet; PD, Parkinson’s disease; UPDRS, United Parkinson’s Disease Rating Scale; DBS, deep brain stimulation.

Implantation of bilateral (seven cases) or unilateral (two cases) STN DBS electrodes was performed in the patients. The DBS electrodes used were model 3389 (Medtronic Neurological Division, Minnesota, USA), with four platinum–iridium cylindrical contacts (1.27 mm in diameter and 1.5 mm in length) and a centre-to-centre separation of 2 mm. Briefly, DBS electrodes were aimed at the centre of the STN after prior identification of this structure using pre-operative magnetic resonance imaging (MRI). The MRI software provided the distance between the anterior and posterior commissures in relation to the centre of a stereotactic frame fixed to the skull, to enable digitized atlas images (Schaltenbrand & Wahren, 1977) of the brain to be adjusted to coincide with the intercommissural line of the patient. Adjustments to the intended coordinates were made in accordance with the direct visualization of the STN on individual stereotactic MRI (Hariz *et al.*, 2003). Furthermore, targeting was confirmed by intra-operative electrical stimulation (all cases), micro-electrode recordings (cases 1, 3, 4, 5 and 7) and post-operative stereotactic MRI or computed tomography with co-registration to pre-operative MRI. Additional evidence in support of accurate targeting was provided by the clinical response during long-term follow-up. Mean pre-operative United Parkinson’s Disease Rating Scale scores off levodopa (33 ± 8) decreased (*P*=0.003) after high-frequency stimulation (13 ± 3) in the seven patients in whom 6 month post-operative clinical assessments had been performed. Contact 0 of the inserted DBS electrode was the lowermost, and contact 3 was the uppermost. The theoretical coordinates at the tip of contact 0 were 10–12 mm from the midline, 0–2 mm behind the midcommissural point and 4–5 mm below the anterior–posterior commissural line.

### Experimental protocol

Patients sat at rest while single-pulse TMS (30–50 stimuli) was delivered every 5 s over one (*n*=3 cases) or both (*n*=6) primary motor (M1) cortices in turn at intensities of 95% and 115% resting motor threshold (RMT) of the contralateral first dorsal interossius (FDI). In two cases (cases 1 and 3), TMS was delivered at 85% RMT instead of 95% RMT to ensure that no descending volleys were induced in the corticospinal tract. TMS was also delivered over the supplementary motor (SMA) at 95% and 115% RMT for the right FDI so that the stimulation intensity was kept constant for this site and the left M1. Single-pulse TMS has been applied to patients with implanted stimulators and is considered to be safe (Kumar *et al.*, 1999; Kühn *et al.*, 2002). All patients had taken their usual medication on the day of study. TMS was carried out with a Novametrix Magstim 200 stimulator or a Magstim 200 Monopulse stimulator (Magstim, Whitland, UK). Monophasic pulses were delivered through a figure-of-eight coil (70 mm diameter) held in a posterior–anterior direction over each cortical area. For M1 stimulation, the coil was placed tangentially against the scalp with the handle pointing backwards and laterally at a 45° angle away from the midline, whereas for SMA stimulation, the coil was placed over the nearest scalp site anterior to *Cz* of the 10–20 system that evoked no twitch in the tibialis anterior muscles (Ziemann *et al.*, 1997; Gregori *et al.*, 2005) with the handle pointing directly backwards. This procedure was chosen because leg representations in M1 lie adjacent to the SMA and at a similar depth within the interhemispheric fissure. A sham condition was also performed in four of the patients (cases 1, 3, 6 and 8; *n*=8 sides) at rest where TMS was delivered at 115% RMT at an angle away from the head, but over the DBS leads. To control for direct muscle or peripheral nerve stimulation at the scalp, an additional sham condition was performed in cases 1 and 3, where TMS at 115% RMT was applied over the occipito-parietal cortex. All cortical sites were marked using a red chinagraph pencil to ensure consistent stimulation of these sites in all stimulation blocks.

Prior to the experiment proper, the coil was moved in 0.5 cm steps around the presumed motor area to determine the optimal site for M1 stimulation, while motor evoked potentials (MEPs) were recorded from the FDI of the contralateral hand using bipolar Ag/AgCl surface electrodes. Individual RMT was defined as the minimum intensity required to evoke MEPs of at least 50 μV in five out of 10 consecutive trials. Electromyographic (EMG) signals were amplified (×1000) and bandpass filtered at 16–500 Hz using a D360 amplifier (Digitimer Ltd, Welwyn Garden City, UK) and digitized at a rate of 1000 Hz through a 1401 A–D converter (Cambridge Electronic Design, Cambridge, UK) onto a computer using Signal3 software (Cambridge Electronic Design).

Continuous recordings were made from the STN with patients at rest for ∼100 s before and then throughout the stimulation. In cases 2, 6, 8 and 9 from London, local field potentials (LFPs) were recorded monopolarly from each contact of each DBS electrode, referenced to linked ears and relayed to a TMS-compatible amplifier (Nexstim Ltd, Helsinki, Finland) using a custom-made adapter. LFPs were amplified (×2000), bandpass filtered at 0.1–350 Hz, and digitized onto a built-in computer using eXimia software (Nexstim Ltd), with a sampling rate of 1450 Hz. EMG signals were not recorded during the experiment proper, as the Nexstim amplifier system prohibited simultaneous recording of EMG signals, and the use of additional amplifiers introduced excessive artefact. Nevertheless, the absence or presence of muscle twitches during LFP recording at the previously established subthreshold and suprathreshold intensities was reliably confirmed by visual inspection throughout all stimulation blocks. The TMS stimulus was recorded as a series of markers in a separate trigger channel. In case 1 from Berlin and cases 3, 4, 5 and 7 from Rome, LFPs were recorded bipolarly from the adjacent contacts of each electrode (0–1, 1–2, 2–3). The signals were amplified (×100 000) and bandpass filtered (1–1000 Hz) using a D360 amplifier (Digitimer Ltd) and then digitized through a 1401 A–D converter (Cambridge Electronic Design) and sampled at a rate of 1–2 kHz onto a computer using Spike5 software (Cambridge Electronic Design). The stimulus artefact recorded in each trial for these cases was used to determine stimulus onset. EMG activity was recorded throughout the experiments in cases 1 and 3 where TMS was delivered at 85% RMT and 115% RMT.

### Analysis

Recordings from the Nexstim system were converted into Spike5 format using a custom-made Spike2 script. In the monopolar recordings, data from adjacent contacts were then subtracted to produce bipolar montages (0–1, 1–2, 2–3) in separate channels for each STN. The first analysis step was to identify the bipolar contact that recorded the highest power in the frequency range of interest, from 13 to 35 Hz, on each side in each patient. Spectral power was calculated with 1 Hz resolution using the fast Fourier transform in Spike5 to determine the dominant frequency ranges for each STN contact pair in recordings taken from the patients at rest without TMS. The contact pair with the highest power in the 13–35 Hz range was determined for each side in each patient and used for subsequent analyses (five at STN 01; seven at STN 12; four at STN 23).

Raw data from the selected bipolar contacts were then averaged to the TMS triggers for each condition to examine event-related potentials (ERPs). The stimulus artefact was not removed from the data for this analysis step. Averages were inspected for phase reversals, and the latencies of consistent evoked potentials were noted. Note that initial ERP components were obscured by stimulus artefact (see Results). For frequency analysis, the stimulus artefact was removed using a Spike script (Artrem v.2) with no more than 10 ms of data removed from each trial in data recorded with the Nexstim system, whereas longer-duration artefacts recorded with the D360 system (cases 1, 3, 4, 5 and 7) required up to 80 ms of data to be removed from each trial. The power spectra of the selected bipolar contacts were then inspected to determine the specific frequency of the bin with the maximal value in the 13–35 Hz range, as beta activity is believed to be maximal in the STN. Individual beta peaks for each patient’s STN were defined as five contiguous 1 Hz bins with a centre frequency corresponding to the peak frequency as determined above. Where there was more than one peak in the beta band, the peak with the highest power was used. Data within these individualized ranges as well as within the alpha range (8–12 Hz) were later extracted from matrix files generated from the original signals using Matlab 7.0.1 (The Mathworks Ltd, Cambridge, UK) for further analysis (see below). In two STNs, there was no discrete beta peak over 13–35 Hz, so data within a 5 Hz band were taken from the middle of this frequency band.

The raw data files containing concatenated trials 4 s long with a 2 s trigger offset were further analysed using a custom-made Matlab script calling functions from eeglab (v. 5.03, http://www.sccn.ucsd.edu/eeglab/) and FieldTrip (http://www.ru.nl/fcdonders/fieldtrip) toolboxes. The trials were visually inspected for mains or movement artefacts, and affected trials were rejected. The data were resampled at 256 Hz after first low-pass filtering at 100 Hz to avoid aliasing. Power line noise at 50 Hz was removed using a discrete Fourier transform filter (Schoffelen *et al.*, 2005). Event-related power was calculated for each epoch for preselected bipolar channels over the range 1–99 Hz with 2.8 Hz resolution using a Hanning taper. The time–frequency decomposition was performed with a time window of 350 ms shifted in steps of 100 ms. Trials were averaged to produce a matrix of event-related power values. Matrix plots were then constructed of the percentage changes in power from a baseline period of 2–0.1 s before the trigger and values of event-related desynchronization (ERD: power decrease) or synchronization (ERS: power increase), thresholded according to the confidence limits of this baseline period.

**Fig. 2:**
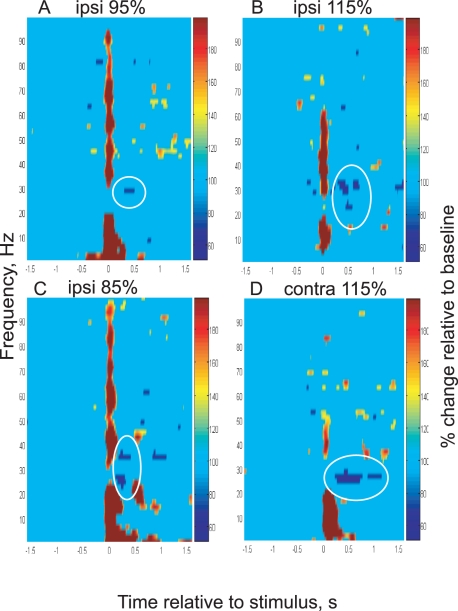
Changes in power in the right subthalamic nucleus over the frequency range 1–99 Hz induced by: (A) ipsilateral primary motor area (M1) transcranial magnetic stimulation (TMS) at 95% resting motor threshold (RMT) in case 8; (B) ipsilateral M1 TMS at 115% RMT in case 2; (C) ipsilateral M1 TMS at 85% RMT in case 3; and (D) contralateral M1 TMS at 115% RMT in case 8. Data have been thresholded according to 95% confidence limits of the baseline. Stimulus is at 0. White circles indicate beta event-related desynchronization.

For each condition, the mean power in the alpha and individualized beta bands was calculated for a baseline period from 1590 to 1230 ms before the stimulus and for a corresponding 360 ms period from 190 to 550 ms post-TMS. The latter period was chosen from the time period for which data fell below the 95% confidence limits in the grand average of beta activity following ipsilateral stimulation at 115% RMT. Data were analysed using repeated measures analysis of variance (anova) and *post hoc* paired *t*-tests (*P*<0.05; spss 12.0.1, Woking, UK). First, data from M1 stimulation were submitted to a three-way repeated measures anova with factors time (pre-TMS vs. post-TMS), laterality (ipsilateral vs. contralateral TMS) and intensity (85/95% vs. 115% RMT). Data relating to subthreshold intensities were combined, due to the similarity of responses detected at these intensities (Fig. 2A and C). Two separate two-way anovas were then performed with factors time (two levels) and site (ipsilateral, contralateral, SMA) for each intensity. A final two-way anova with factors time (two levels) and site (ipsilateral, contralateral, leads) was used to establish whether stimulation over the DBS leads at 115% RMT could also induce an effect. For each anova, Mauchley’s test of sphericity was used to assess whether the *P*-values required adjustment, but no correction for non-sphericity was necessary.

Finally, the focality of the beta band LFP signals was investigated by cross-correlation of the 13–35 Hz bandpass filtered waveforms from adjacent contact pairs (e.g. 01 and 12; 12 and 23) in Spike. Peak negative cross-correlations at time zero ± 5 ms were assumed to indicate polarity reversal at the contact held in common by the respective two contact pairs (Brown *et al.*, 2001; Kühn *et al.*, 2004). In addition, the ERD was calculated as above (i.e. for a 360 ms period from 190 to 550 ms post-TMS) separately for each bipolar pair to establish the pair with the maximum ERD and the percentage by which the ERD fell at remaining bipolar contacts relative to this maximum value.

## Results

### TMS-evoked MEPs and ERPs

**Fig. 1:**
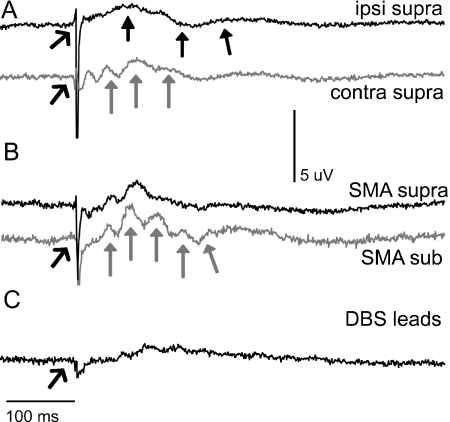
Event-related potentials in Parkinson’s disease patients. (A) Grand average of subthalamic nucleus (STN) activity following transcranial magnetic stimulation (TMS) delivered to the ipsilateral (black, *n*=14 STNs) and contralateral (grey, n=13 STNs) primary motor area at 115% resting motor threshold (RMT). (B) Grand average of STN activity following TMS over the supplementary motor area (SMA) at 115% RMT (black, *n*=10 STNs) and at 95% RMT (grey, *n*=6). (C) Grand average of STN activity following TMS over deep brain stimulation (DBS) leads (*n*=8 STNs) at 115% RMT. Open arrows denote stimulus artefact. Closed arrows denote evoked potentials. Data from left and right STNs have been combined.

**Fig. 3:**
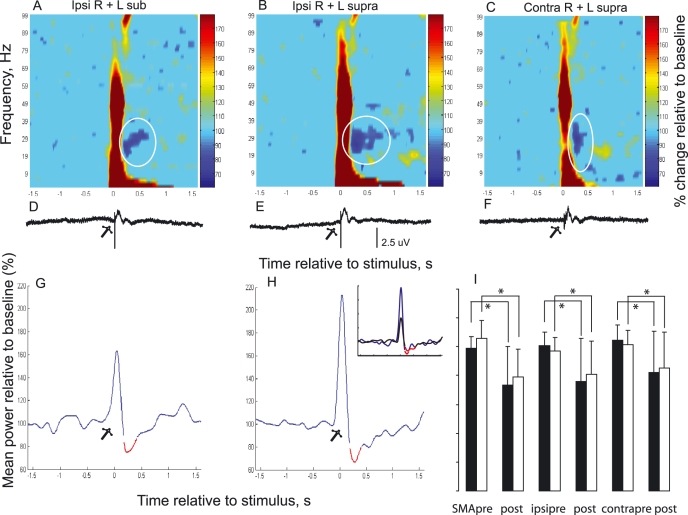
Group mean changes in subthalamic nucleus (STN) power induced by transcranial magnetic stimulation (TMS) over the primary motor area (M1). Mean changes in power in the STN over the frequency range 1–99 Hz induced by: (A) ipsilateral M1 TMS at 85%/95% resting motor threshold (RMT) (*n*=14 STNs); (B) ipsilateral M1 TMS at 115% RMT (*n*=14 STNs); and (C) contralateral M1 TMS at 115% RMT (*n*=13 STNs). White circles indicate beta event-related desynchronization. (D–F) Grand means of local field potential data averaged to TMS from the same dataset as shown in A–C, with an identical time scale. (G and H) Grand averages of individualized beta activity from same dataset as in A and B. (H inset) Averaged beta response to ipsilateral suprathreshold stimulation from patients recorded in London (black) and Rome (blue). See also supplementary Fig. S2. Red lines indicate values significantly smaller than the lower 95% confidence limit. All changes are expressed as percentages of the baseline mean, taken 1.59–0 s prior to stimulation. Data from both STN sides have been combined in all matrix and line plots. Stimulus at 0 (open arrows). (I) Mean beta power (±SD) in STNs before (1.59–1.23 s) and after (0.19–0.55 s) suprathreshold (filled columns) and subthreshold (open columns) TMS at different cortical sites. Asterisks denote significant differences (*P*<0.05).

MEPs were evoked in the contralateral FDI by suprathreshold TMS over M1, but not by subthreshold TMS at 85% over this area or by suprathreshold TMS delivered over the occipito-parietal cortex in cases 1 and 3, in whom EMG signals were simultaneously recorded with TMS (see Supplementary material, Fig. S1). LFP data averaged in the PD cases to subthreshold and suprathreshold stimulation delivered over the ipsilateral motor cortex revealed a positive deflection peaking at ∼75 ms, a negative deflection at ∼170 ms, and then another positive deflection at ∼210 ms in each patient (Fig. 1A, black closed arrows). Shorter-latency components were inconsistent and/or obscured by stimulus artefact (Fig. 1, open arrows). Evoked potentials could also be seen following subthreshold and suprathreshold TMS applied to the SMA, although in this case the positive wave peaking at ∼75 ms comprised a short series of subcomponents repeating every 25 ms (Fig. 1A and B, grey closed arrows). There was little difference between the potentials evoked by subthreshold or suprathreshold TMS (Figs 1B and 3D and E). No consistent potentials were found following stimulation over the DBS leads, although a large non-saturating stimulus artefact in one individual slightly distorted the mean response (Fig. 1C). Some small inconsistent short-latency potentials were detected below 100 ms together with a late deflection ∼350 ms after occipito-parietal stimulation (data not shown), but none were found to correspond to those evoked by TMS over motor areas.

### TMS-evoked ERD

**Fig. 4:**
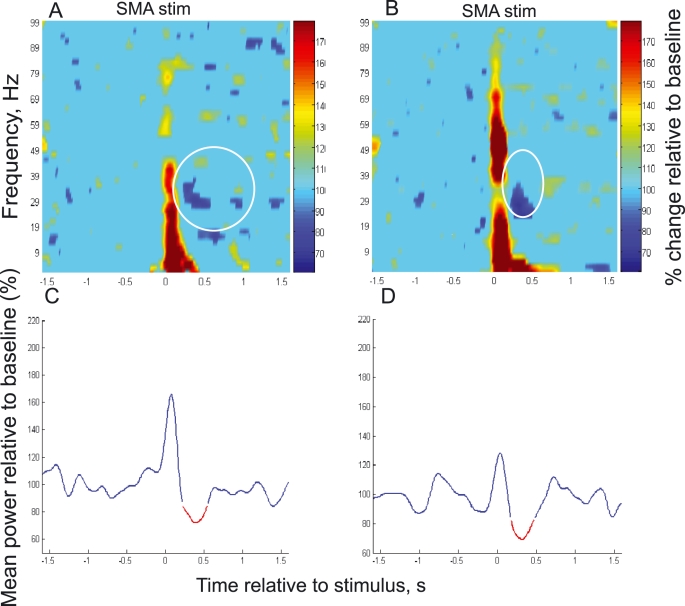
Changes in subthalamic nucleus (STN) power induced by transcranial magnetic stimulation over the supplementary motor area (SMA) in Parkinson’s disease patients. Group mean colour matrix in response to stimulation at: (A) 95% resting motor threshold (RMT) (*n*=6 STNs) delivered over the SMA; (B) 115% RMT (*n*=10 STNs) over the SMA. White circles indicate beta event-related desynchronization. (C and D) Grand average of individualized beta activity from the same dataset as shown in A and B. Red lines indicate values significantly smaller than the lower 95% confidence limit. All changes are expressed as percentages of the baseline mean, taken 1.59–0 s prior to stimulation with data from both STN sides combined. Stimulus at 0 in all plots.

Discrete peaks in LFP autospectra were detected over 13–35 Hz at rest prior to stimulation in all but two STNs (cases 2 and 6). The mean peak frequency was 23 ± 6 Hz. Matrices of TMS-induced power change were plotted for each STN. Despite artefact removal, there was still some residual power increase around the trigger, which tended to last longest at low frequencies, particularly in the alpha band (Figs 2–4), and most likely represented a combination of effects from evoked potentials in the LFP (Figs 1 and 3) and residual stimulus artefact which varied between sides. It was not possible to disambiguate the contribution of each to this initial increase in power.

An ERD (LFP power suppression) within 13–35 Hz followed the major power increase, although in some individuals it began while power was still elevated at frequencies under 10 Hz.

The ERD was evident in matrices from individual patients in response to subthreshold and suprathreshold ipsilateral M1 TMS (Fig. 2A–C) and contralateral M1 stimulation (Fig. 2D), and was consistently observed in patients from the different surgical centres (Figs 2 and 3H, inset; supplementary Fig. S2). It was also seen in grand average matrices and time series of averaged individualized beta activity in response to subthreshold ipsilateral M1 stimulation (Fig. 3A and G), suprathreshold ipsilateral M1 stimulation (Fig. 3B and H), and suprathreshold contralateral M1 stimulation (Fig. 3C). There was no obvious relationship between the grand average ERPs and grand average ERD over 13–35 Hz (Fig. 3A–F), with, in particular, the ERD following TMS over the ipsilateral M1 outlasting the ERP. The grand average individualized beta ERD in response to suprathreshold ipsilateral M1 stimulation began 190 ms after the stimulation, had its minimum value at 270 ms, and returned to baseline levels at 550 ms (Fig. 3H). An anova (time, laterality, intensity) revealed a main effect of time (time period pre-TMS and post-TMS: Fig. 3I; *F*_1,12_=10.68, *P*=0.007), and of laterality (*F*_1,12_=6.19, *P*=0.03), as the ipsilateral ERD was larger and of longer duration than that occurring contralaterally (Fig. 3B and C). There was no effect of TMS intensity (*F*_1,12_=0.009, *P*=0.92), and nor were there any interactions between any of the factors. However, there was no ERD observed in the alpha range, although a similar anova of alpha activity revealed an effect of time (*F*_1,12_=18.49, *P*=0.001), but this was due to an increase in alpha activity that was at least partly related to stimulus artefact and evoked potentials. There was no effect of laterality or intensity or any interactions between factors for alpha activity.

The grand average matrices and time series of averaged individualized beta activity in response to subthreshold (Fig. 4A and C) and suprathreshold (Fig. 4B and D) stimulation over the SMA also confirmed ERDs in the beta bands but not alpha bands. Two separate anovas of beta activity (time, site) showed that there was an effect of time (time period pre-TMS and post-TMS) at 115% RMT (*F*_1,8_=10.57, *P*=0.01) and almost at 95% RMT (*F*_1,5_=6.5, *P*=0.05). However, there was no difference across sites (ipsilateral, contralateral, SMA) and no interaction between time and site for either intensity. Overall, oscillatory LFP activity in the beta band was suppressed to 80 ± 26% of baseline values (averaged across all cortical sites and stimulation intensities). anovas of alpha activity revealed no changes related to time or site at either intensity.

### Sham TMS

**Fig. 5:**
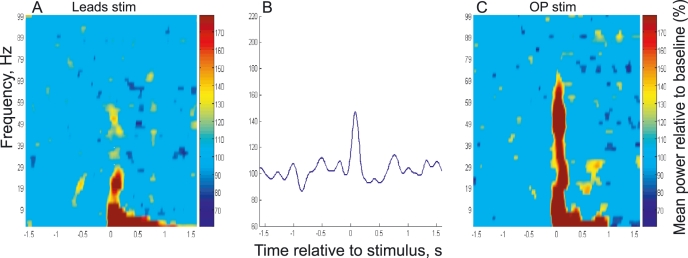
Sham transcranial magnetic stimulation (TMS). (A) Mean changes in subthalamic nucleus (STN) power over the frequency range 1–99 Hz induced by suprathreshold TMS (115% resting motor threshold) delivered over the deep brain stimulation (DBS) leads. Data have been thresholded according to 95% confidence limits. (B) Grand average of individualized beta activity following suprathreshold TMS delivered over the DBS leads. No values fell below the lower 95% confidence limit. All changes are expressed as percentages of the baseline mean, taken 1.59–0 s prior to stimulation. Data from both STN sides have been combined (*n*=8 STNs). (C) Mean changes in STN power over the frequency range 1–99 Hz induced by suprathreshold TMS delivered over the occipito-parietal (OP) cortex (*n*=4 STNs). Data have been thresholded according to 95% confidence limits.

Suprathreshold TMS over the DBS leads alone failed to induce a consistent alpha or beta ERD in the STN, as evidenced by the group average matrix and time series of averaged individualized beta activity (Fig. 5A and B). The increase in power across low and high frequencies around the time of the stimulus was somewhat reduced as compared to that seen during stimulation over the M1 or SMA (Figs 2–4), suggesting that some of this increase following cortical TMS was related to the evoked potentials. An anova (time, pre-TMS and post-TMS, site, ipsilateral cortex, contralateral cortex and over DBS leads) of beta power revealed that there was a main effect of time (*F*_1,6_=10.85, *P*=0.02), but no effect of site, although there was an interaction between time and site (*F*_1,6_=4.4, *P*=0.04), due to the decrease in beta power following ipsilateral and contralateral cortical TMS (paired *t*-tests; ipsilateral, *P*=0.003; contralateral, *P*=0.014) that was not seen after stimulation over the DBS leads (paired *t*-test; *P*=0.23). There was also an effect of time for alpha activity as before (*F*_1,6_=10.21, *P*=0.02), but no effect of site or any interaction between the two. Suprathreshold TMS over the occipito-parietal cortex induced a short latency power increase around the trigger, but failed to induce an alpha or beta ERD (Fig. 5C).

### Focality of ERPs and ERD

Averaged LFP recordings showed phase reversals of ERPs in 75% of STN sides, whereas power spectra at rest revealed that 13–35 Hz power was greater at one out of the three contact pairs in all but two STN sides (87.5%), falling by 61.3 ± 29% at remaining contact pairs. Cross-correlation of the 13–35 Hz bandpass-filtered waveforms from adjacent contact pairs confirmed polarity reversals in 13 out of 16 (81.3%) of STN sides. These results are highly suggestive of a focal origin of the evoked activity and oscillatory changes. Moreover, the ERD was always greatest at one contact pair on each side. The ERD at the remaining contact pairs was reduced by 81.2 ± 84% of the maximum ERD for that side following suprathreshold stimulation over the left M1, and 295.3 ± 805.7% (e.g. a relative but very variable ERS at other contact pairs) after suprathreshold TMS over the right M1. The observed maximum ERD occurred at the same bipolar contacts in 75% of sides, regardless of which cortical side was stimulated.

## Discussion

These results demonstrate that single-pulse TMS can evoke a delayed suppression of beta power in the human STN. This pattern was seen with subthreshold as well as suprathreshold stimulation, suggesting that changes in oscillatory activity in the STN were centrally driven and not due to peripheral afference secondary to evoked muscle responses. The power suppression could be elicited by stimulation over the ipsilateral motor cortex, SMA and contralateral motor cortex, but not by stimulation over the DBS leads or occipito-parietal area. Moreover, the stimulation-induced ERD was frequency selective and not seen in the alpha band.

### Experimental limitations

There were, however, some limitations to our experimental approach. It could be argued that bilateral responses were generated as a result of direct activation in both hemispheres by small currents induced in the DBS leads by TMS (Kumar *et al.*, 1999; Kühn *et al.*, 2002; Hidding *et al.*, 2006). However, the long latency of the beta ERD would be against this. Furthermore, suprathreshold stimulation over the DBS leads failed to induce a beta ERD. In addition, the beta ERD was sometimes briefer after contralateral stimulation than after ipsilateral stimulation (compare Fig. 3C with Fig. 3B), a feature that would be inconsistent with stimulation due to the (bilateral) induction of current flow in the DBS leads. The absence of a beta ERD following sham stimulation over the DBS leads or occipito-parietal cortex also helps to exclude the possibility that the subthalamic ERD was related to auditory stimulation through the click of the TMS coil or direct muscle/peripheral nerve stimulation of the scalp.

Could the oscillatory changes have been related to peripheral re-afference? Although an ERD was observed following TMS at 95% RMT, this intensity of stimulation could still have conceivably evoked occasional muscle responses even though none were detected visually. However, a significant ERD could still be observed following TMS at 85% RMT over both ipsilateral and contralateral motor areas, in the two subjects so tested, and here simultaneous EMG recordings were performed, which excluded any muscle responses. It seems unlikely, therefore, that somatosensory input could have contributed to the observed power changes.

A related possibility is that the beta suppression might have been secondary to a TMS-induced suppression of rest tremor. However, although a relationship between beta activity and tremor has been reported (Levy *et al.*, 2000), doubt has since been cast on the validity of this observation by the same group (Weinberger *et al.*, 2006). Moreover, we recorded our patients in the on-drug state so that rest tremor was suppressed.

Without histological verification of electrode site, placement in the STN should be considered presumptive. Nevertheless, surgical coordinates and clinical efficacy were consistent with placement of one or more DBS electrode contacts in the STN, and the finding of LFP spectral power in the beta band has been previously reported to be of localizing value in identifying the STN (Brown *et al.*, 2001; Williams *et al.*, 2002; Kühn *et al.*, 2004, 2005; Sharott *et al.*, 2005; Chen *et al.*, 2006; Weinberger *et al.*, 2006; Trottenberg *et al.*, 2007). The possibility that the ERD phenomenon was due to the volume conduction of modulated cortical activity must also be considered. The bipolar montages used limit the possibility of contamination of depth signals by cortical electroencephalography (EEG). Moreover, LFP recordings from adjacent DBS electrode contact pairs showed a dominance of beta activity and its suppression at one contact pair in all but two STN sides and polarity reversals in 81% of STN sides, suggestive of a focal origin.

Importantly, we did not perform simultaneous EEG, which would have been advantageous in determining cortical contributions to subcortical power changes. EEG recordings were not possible because of the nature of scalp bandages. Finally, it is worth remembering that the pattern of activity observed in the STN of PD patients might not reflect that occurring in healthy individuals. However, all patients had taken their usual anti-parkinsonian medication, so that their dopaminergic deficiency was relatively normalized.

### Origin of the LFP power changes at the level of the STN

Paus *et al.* (2001) and Fuggetta *et al.* (2005) have reported that single-pulse TMS over M1 in healthy humans evokes a period of increased activity in the beta range at the cortical level, and Magill *et al.* (2006) similarly described a synchronization of activity in this frequency in the rat STN after cortical stimulation. It is possible that the above contributed to the early increase in power that we found in the STN around the time of the stimulus, although the initial power increase may also be explained by residual stimulus artefact and by evoked potentials in the LFP.

There are several possible explanations for the power suppression in the beta band following TMS. The latency and duration of the ERD were relatively long. This, together with other reasons outlined above, suggests that the ERD was not simply the product of current flow in the DBS electrodes induced by TMS. This leaves three principal possibilities, which need not be mutually exclusive. First, the previously reported increase in cortical beta activity following TMS may be followed by a temporary rebound suppression, with this then being reflected in the STN, due to withdrawal of the beta drive from the cortex during this period. Second, the descending volley or volleys precipitated in the bilateral cortico-subthalamic and cortico-striatal projections from the motor cortex and SMA (Nambu *et al.*, 1997; Takada *et al.*, 1998; Inase *et al.*, 1999) by TMS may themselves modulate beta activity that might be generated in the basal ganglia. Here, in order to account for the delayed ERD in the STN, we would have to posit that re-entrant cortico-thalamo-cortical pathways are involved or that descending volleys have both synchronizing (Magill *et al.*, 2006) and desynchronizing effects in the basal ganglia and that, in the alert subject, the latter lasts longer, giving the appearance of a delayed ERD. Alternatively, the cortically induced beta ERS might be propagated to subcortical levels and obscure the earliest phase of the beta ERD in the STN. Concurrent EEG recordings would have been desirable in this regard. Finally, we should consider one other possibility. Subthreshold repetitive TMS has been shown to elicit a focal release of endogenous dopamine in the striatum of rats (Keck *et al.*, 2002; Kanno *et al.*, 2004; Funamizu *et al.*, 2005) and humans (Strafella *et al.*, 2001, 2003; Pogarell *et al.*, 2006), and dopamine is known to suppress beta oscillations in the basal ganglia, including the STN [reviewed in Brown & Williams (2005)]. An upregulation of dopamine release induced by TMS could therefore contribute to the ERD.

## Conclusion

We have demonstrated a temporary suppression of beta activity in the human STN following cortical TMS, in line with the suppression of abnormally synchronized oscillations, albeit at lower frequencies, noted by Drouot *et al.* (2004) in the primate internal globus pallidus and STN during repetitive cortical stimulation. Under physiological conditions, the cortical output precipitated by TMS might be paralleled by phasic movement-related corollary discharge from the cortex to the STN (Marsden *et al.*, 2001; Magill *et al.*, 2006). Accordingly, the suppression of beta band activity in the basal ganglia in response to cortical output might help to facilitate motor-related processing in these nuclei (Courtemanche *et al.*, 2003).

Our results may also be relevant to the therapeutic effects that are being reported following cortical stimulation in PD. The suppression of exaggerated beta synchrony in basal ganglia–cortical loops has been suggested as a basic mechanism of action of both levodopa and functional neurosurgery (Hammond *et al.*, 2007). We found that the suppression of beta band activity lasted ∼400 ms following single-pulse cortical stimulation, suggesting that regular stimulation by either repetitive TMS or epidural electrical stimulation at frequencies above ∼5 Hz might lead to persistent suppression of subcortical beta activity during stimulation, with an associated amelioration of parkinsonism. In line with this, both the suppression of beta activity in the STN in our study and improvements in motor performance reported in monkeys following unilateral extradural motor cortical stimulation (Drouot *et al.*, 2004) were bilateral. The next steps are to establish the origin of the changes in beta activity in the STN (withdrawal of cortical oscillations or suppression of subcortical oscillations), determine whether such activity in the basal ganglia can be persistently suppressed during continuous epidural stimulation or repetitive TMS in patients with PD, and determine whether this suppression is dopamine-dependent.

## Abbreviations

DBS: deep brain stimulation
EEG: electroencephalography
EMG: electromyography
ERD: event-related desynchronization
ERP: event-related potential
ERS: event-related synchronization
FDI: first dorsal interossius
LFP: local field potential
M1: primary motor area
MEP: motor evoked potential
MRI: magnetic resonance imaging
PD: Parkinson’s disease
RMT: resting motor threshold
SMA: supplementary motor area
STN: subthalamic nucleus
TMS: transcranial magnetic stimulation
UPDRS: United Parkinson’s Disease Rating Scale

## Supplementary material

The following supplementary material may be found on http://www.blackwell-synergy.com

Fig. S1. Motor evoked potential response to transcranial magnetic stimulation.

Fig. S2. Group mean changes in subthalamic nucleus beta power following transcranial magnetic stimulation.
