# Supplementary figures and images for: Suppression of beta oscillations in the subthalamic nucleus following cortical stimulation in humans
Source: Eur J Neurosci. 2008 Oct;28(8):1686–95. doi: 10.1111/j.1460-9568.2008.06363.x (PMC2695156; doi:10.1111/j.1460-9568.2008.06363.x)

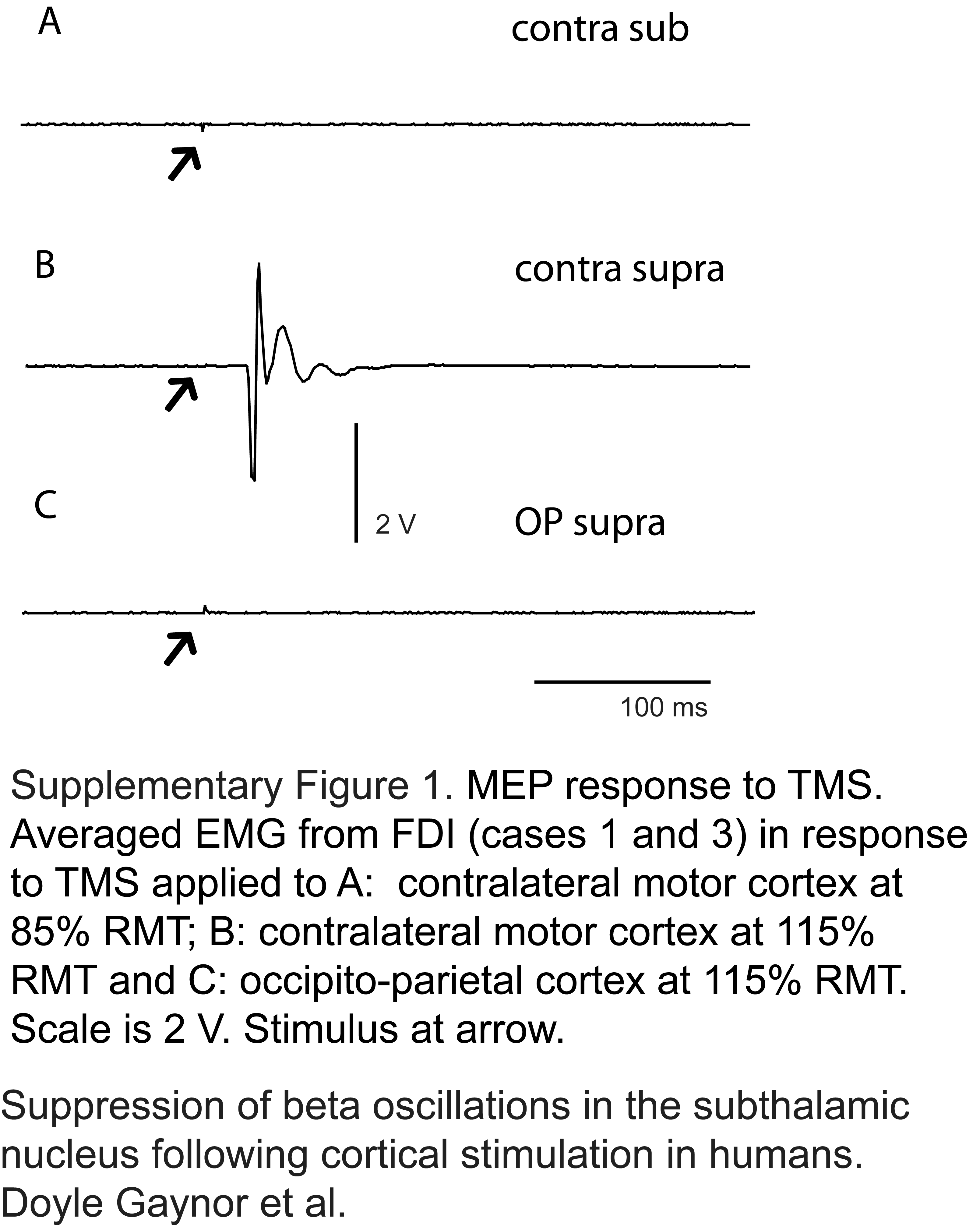

Supplement: Supplementary file 1 [file ejn0028-1686-SD1.tif]

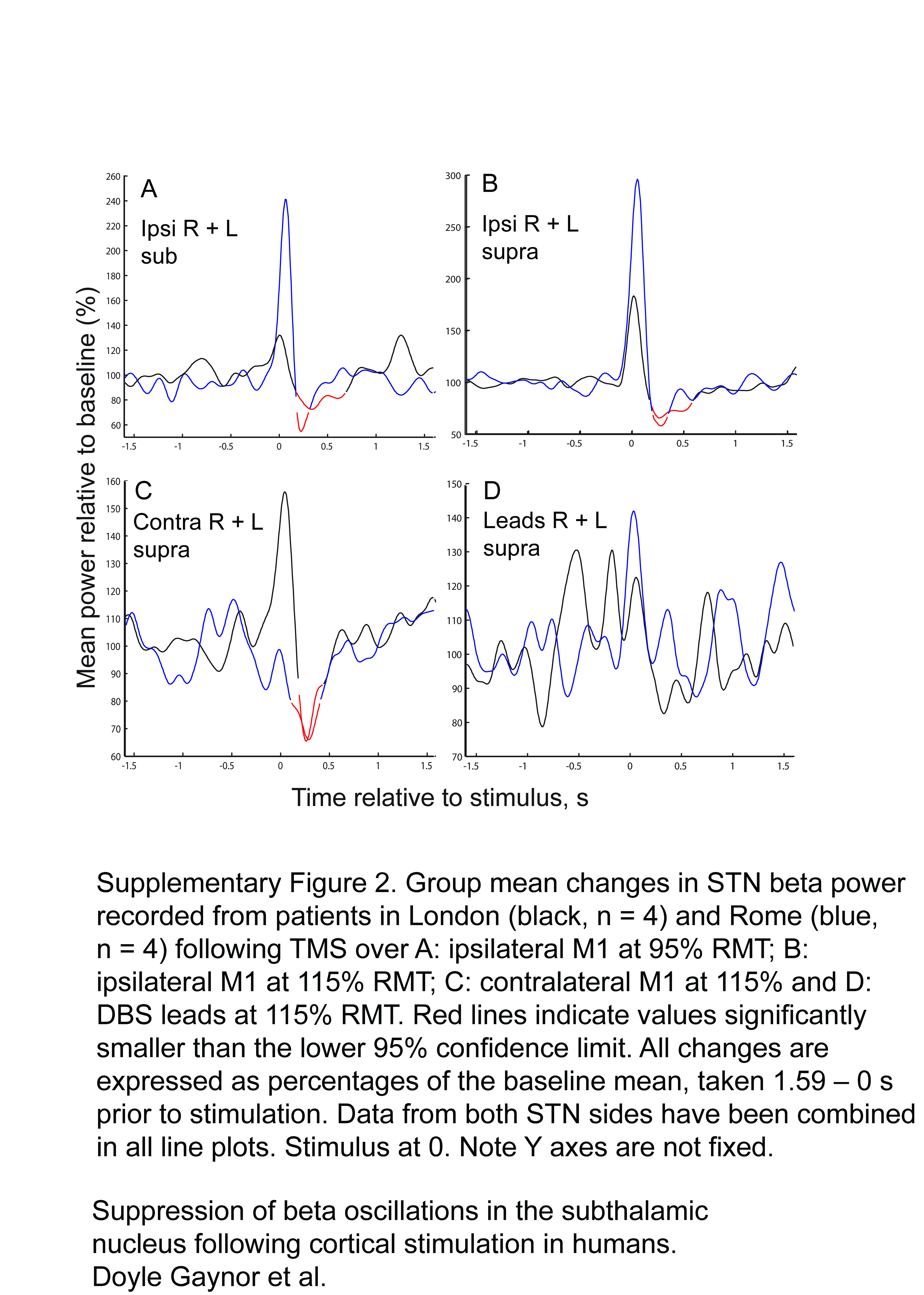

Supplement: Supplementary file 2 [file ejn0028-1686-SD2.tif]
